# ROS-mediated Different Homeostasis of Murine Corneal Epithelial Progenitor Cell Line under Oxidative Stress

**DOI:** 10.1038/srep36481

**Published:** 2016-11-02

**Authors:** Jing Zhou, Lianping Ge, Changkai Jia, Xiling Zheng, Huixia Cui, Rongrong Zong, Xiaorui Bao, Yuanyuan Yin, Jian-xing Ma, Wei Li, Zuguo Liu, Yueping Zhou

**Affiliations:** 1 Eye Institute of Xiamen University, Fujian Provincial Key Laboratory of Ophthalmology and Visual Science, Xiamen, Fujian, 361005, China; 2 Department of Physiology, University of Oklahoma Health Sciences Center, Oklahoma City, Oklahoma 73104, United States of America

## Abstract

The role of ROS in stem cell biology has not been fully illustrated and understood. Here we compared the different responses and investigated the mechanism underlying oxidative stress induced by hydrogen peroxide (H_2_O_2_) between murine corneal epithelial progenitor cell line (TKE2) and mature murine corneal epithelial cells (MCE). TKE2 showed a different homeostasis and strong resistance to H_2_O_2_. TKE2 reduced the production of ROS, inhibited ROS generation enzyme NADPH oxidase 4 (NOX4), and increased dual specificity phosphatase 6 (DUSP6). Furthermore, TKE2 activated nuclear factor (erythroid-derived 2)-like 2 (NRF2) signaling pathway, regulated miR-125B1 and miR-29B1, and elevated levels of antioxidants glutathione S-transferase P (GSTP) and superoxide dismutases (SOD). The association with ROS of the cells was also verified by RNA interference approach and pharmacological antagonization. In addition, TKE2 enhanced the autophagy after exposure to H_2_O_2_. The novel evidence suggests that TKE2 cells have different homeostasis and strong antioxidant properties against oxidative stress via the regulation of ROS formation and pathway.

The research on complex cell properties and sophisticated cellular functions of stem cells from different origins has drawn increasing attention in the field, due to emerging application and transplantation of stem cells. Scientists have focused on the following major characteristics of stem cells in order to elucidate the unique features of stem cells: cell division, cell differentiation, lineage determination, key signaling pathways, the microenvironment requirement and niche^1^,^2^,^3^. The role of reactive oxygen species (ROS) in stem cell biology has become another important research topic of stem cell properties. Although the mechanism underlying the role of ROS in stem cell biology remains largely unknown, multiple reports demonstrate that stem cells have antioxidant properties^4^,^5^,^6^,^7^, and that oxidative stress status in the environment of stem cells may also affect the cellular functions of transplanted stem cells^8^,^9^,^10^,^11^.

Corneal epithelial stem cells are located in the limbal area, and play vital roles in maintaining normal corneal homeostasis as well as corneal wound healing. Stem cell deficiency is the pathogenesis of some corneal diseases, such as chemical burns and pterygium^12^,^13^. The corneal stem cell preparation, mechanisms, and transplantation have become interesting topics of research for scientists and ophthalmologists^14^,^15^. In the past few years, Kawakita *et al*. developed a murine corneal epithelial progenitor cell line, called TKE2^16^, which manifests characteristics of stem cells; for example, cell self renewal and the expression of stem cell associated markers, such as ATP-Binding Cassette Transporter G2(ABCG2), N-cadherin, and so on^16^,^17^. TKE2 has become a good model to investigate the cell property and mechanism of corneal epithelial stem/progenitor cells^18^.

In the present investigation, we compared the different responses of TKE2 cells and mature murine corneal epithelial cells (MCE) to oxidative stress induced by hydrogen peroxide, and investigated the underlying mechanistic by focusing on the association with the ROS system and signaling pathways.

## Materials and Methods

### Cell Culture

TKE2, a murine limbal/corneal epithelium-derived progenitor cell line, was a kind gift from Dr. Schaffer C.G. Tseng at Ocular Surface Center, Florida^16^. TKE2 cells were cultured in a defined keratinocyte serum-free medium (DKSFM) (Life Technologies Corporation, Carlsbad, CA) supplemented with 10 ng/ml Epidermal Growth Factors (EGF), 1% penicillin/streptomycin. The cells were cultured at 37 °C, 5% CO_2_. In order to induce differentiation of TKE2, 6% FBS was added to conditioned culture medium, and cells were cultured for 2 days prior to the treatment of H_2_O_2_.

Murine corneal epithelial cells (MCE) were prepared from C57 mice (male, 8–10 w) which were provided by Experimental Animal Center of Xiamen University. The preparation procedure was performed according to the ARVO Statement for the Use of Animals in Ophthalmic and Vision Research and the experimental protocol was approved by the Animal Ethics Committee of Xiamen University School of Medicine (approval ID: XMUMC: 2014-12-15). After washing with 1xHBSS containing 1% PS, enucleated eyeballs were digested with 2U dispase II in Cnt20 medium (CELLnTEC, Berne, Switzerland) at 4 °C for about 18 h. The corneal epithelial sheets were then separated with iris spatula and further digested to single cells in 0.05% Trypsin-EDTA at 37 °C for 30 min. The cells were seeded on 35-mm dishes and cultured in 37 °C, 5% CO_2_. MCE cells were cultured in Cnt20 medium supplemented with 10 ng/ml EGF, 1% penicillin/streptomycin. Ultimately, the 15–20 passages of MCE cells were used for the experiments in this study.

When both TKE2 and MCE cells were cultured to approximately 75% confluency, the culture medium was replaced with the conditioned medium containing different concentration of hydrogen peroxide (H_2_O_2_), 40 nM 12-o-tereadecanoylphorbol–13-acetate (TPA), and 10 μM Sulforaphane (SFN) (Sigma, Saint Louis, MO). After treatment for 24 h or at specified time duration, the cells were used for subsequent experiments.

### RNA Isolation and Quantitative RT-PCR

Total RNA and miRNA were extracted from TKE2 and MCE cells using TRIzol reagents (Invitrogen, Carlsbad, CA). Reverse transcription of the extracted RNA into cDNA were conducted using the Transcriptor First-Strand cDNA synthesis kit (TaKaRa, Shiga, Japan) and complementary DNA of miRNA were reverse-transcribed using a TaqMan miRNA Reverse Transcription Kit (Genepharma, Shanghai, China). Quantitative real-time PCR was performed with BIORAD CFX-96 Real Time system with SYBR Premix Ex Taq (TaKaRa). For mature miRNA quantification, the housekeeping gene u6 was served as internal control and the specific probes of miR-125B1 and miR-29B1 were gained from TaqMan miRNA assay kits (Genepharma). Quantitative PCR analysis was performed using TaqMan Universal PCR Master Mix (Genepharma) and a StepOne Real-Time PCR system (Applied Biosystems, Carlsbad, CA). The PCR primers used in the experiment are shown in Supplementary Table S1.

### Immunofluorescent Staining

Immunofluorescent staining was performed as previously described^19^. Primary antibodies (company, catalogue number, and dilution) included anti-ABCG2 (Millipore, MAB4146, 1:50), anti-N-cadherin (Santa Cruz, sc7393, 1:150), anti-3-NT (Abcam, ab61392, 1:150), anti-NOX4 (Abcam, ab60940, 1:150) and anti-NRF2 (Abcam, ab31163, 1:150). Secondary antibodies: donkey-anti-mouse AlexaFluor488, donkey-anti-rabbit AlexaFluor488, were used at a 1:300 dilution.

### Western Blot Analysis

Total cellular proteins were extracted with cold RIPA buffer (Thermo Fisher Scientific, Waltham, MA) and measured by BCA Protein Assay Kit (Thermo Fisher Scientific). Equal amounts of protein were resolved by 10% Tris-glycine SDS polyacrylamide gels and electrotransferred to PVDF membranes (Millipore, Billerica, MA). Western blot analysis was performed as previously described^19^. The following antibodies were used (company, catalogue number, and dilution): anti-N-cadherin (Santa Cruz, sc-7939, 1:500), anti-3-NT (Abcam, ab61392, 1:500), anti-NOX4 (Proteintech, 14347-1-AP, 1:500), anti-DUSP6 (Abcam, ab76310, 1:500), anti-NRF2 (Abcam, ab31163, 1:500), anti-GSTP (Santa Cruz, sc-134469, 1:500), anti-SQSTM1/P62 (Abcam, ab56416, 1:800), anti-Beclin1 (Abcam, ab55878, 1:1200) and anti-LC3B (Abcam, ab63817, 1:1000).

### siRNA transfection

siRNA (Genepharma) was transfected into MCE cells using Lipofectamine 2000 Reagent (Invitrogen) according to the manufacturer’s protocols. Cells were seeded on 12-well plates in advance, and the medium was replaced by a medium without any supplements followed by culture overnight when cells reached 65% confluency. On the day of the experiment, the cells were washed with 1x PBS, then 80 pmol siRNA and 4 μl Lipofectamine 2000 were diluted with DEPC-treated water separately, gently mixed and treated for 6 h. A half of the medium was then changed with the conditioned medium including supplements and the cells were cultured overnight at 37 °C in 5% CO_2_. The cells were used for Western blot and quantitative RT-PCR assay. (Primer for NOX4 siRNA CCAGUGGUUUGCAGAUUUATT (forward), UAAAUCUGCAAACCACUGGTT (reverse)).

### SOD Activity Measurement

When both TKE2 and MCE cells were cultured to approximately 75% confluency, TKE2 and MCE cells were treated with the conditioned medium containing H_2_O_2_ for 24 h, SOD activity was then measured with an assay kit (Jancheng Bioengineering Inc., Nanjing, China) according to the manufacturer’s instructions. It measures the formation of a formazan dye upon reduction of the tetrazolium salt WST-1(2-(4-iodophenyl)-3-(4-nitrophenyl)-5- (2,4-disulfophenyl)-2H-tetrazolium) with superoxide anions. The working solution was incubated for 20 minutes at 37 °C and the absorbance was measured at 450 nm. For the data analysis, the activity was calculated in units and normalized by the amount of protein measured.

### Cell Viability Assay

The cell viability assay was conducted using Cell Counting Kit-8 (Dojindo, Kumamoto, Japan) following the manufacturer’s instructions. After TKE2 and MCE cells were seeded on 96-well plates, and separately treated for 24 h in the conditioned media containing different concentrations of H_2_O_2_. The media were replaced by CCK-8 constituted in culture media, followed by 4 h incubation at 37 °C in the dark, the solution was then detected directly. The absorbance was measured spectrophotometrically at 450 nm with a Bio Tek ELX800 microplate reader (Bio Tek Instruments, Winooski, VT).

### ROS Assay

The productions of ROS in TKE2 and MCE cells were measured following the protocol of the manufacturer. Following overnight treatment of the conditioned medium containing H_2_O_2_, the TKE2 and MCE cells were washed with PBS once, and then were incubated with DCFH-DA (2′,7′-dichlorofluorescein diacetate) (Invitrogen) at 37 °C for 30 minutes. DCF fluorescence distribution of 1 × 10^4^ cells was ultimately detected by flowcytometry at an excitation wavelength of 488 nm and at an emission wavelength of 525 nm.

### Statistical Analysis

All of the data of only two groups were analyzed using Student t-test. All of the data with three or more groups were analyzed using one-way ANOVA, followed by post-hoc Tukey test to compare the difference among the groups. A p value less than 0.05 was considered to be statistically significant.

## Results

### Comparison of Responses to Oxidative Stress Induced by H_2_O_2_

**Figure 1:**
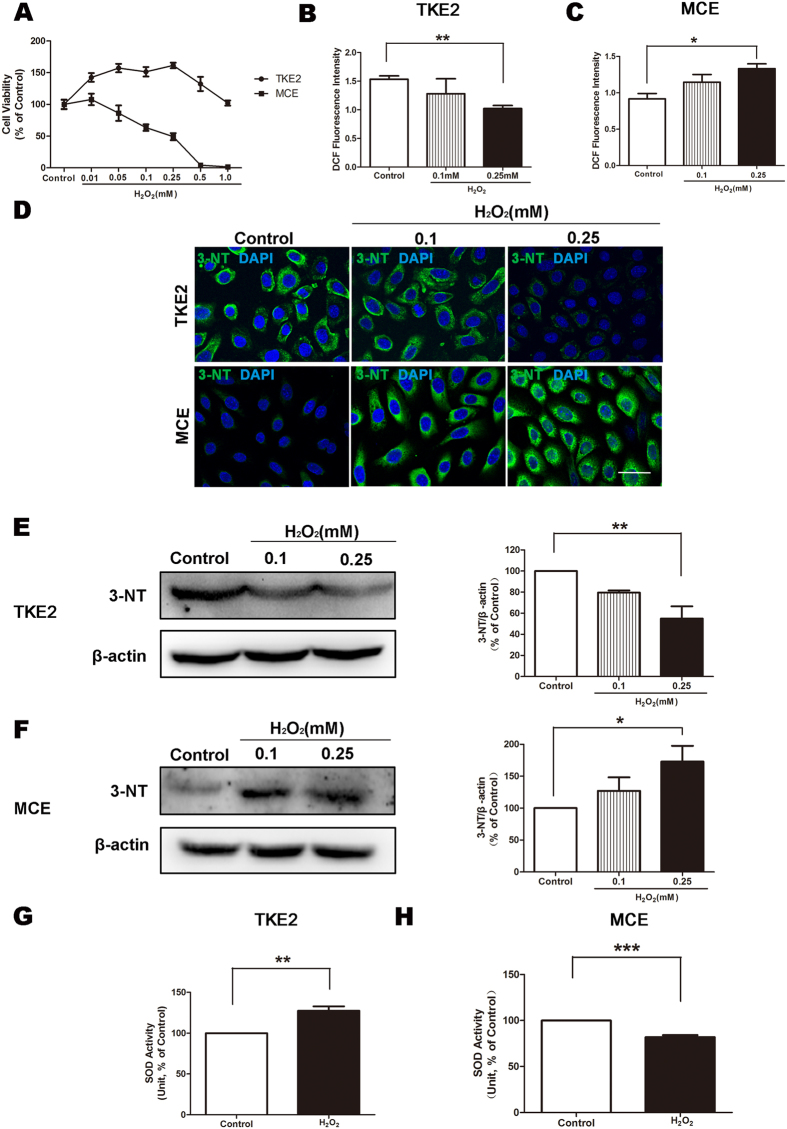
Different ROS-associated responses to oxidative stress induced by H_2_O_2_. (**A**) Cell viability of TKE2 and MCE. (**B**) ROS production was detected by fluorescent DCF assay. DCF fluorescence intensity in TKE2. Data represent mean ± SEM. **P < 0.01. (*n* = 3) **(C)** DCF fluorescence intensity in MCE. Data represent mean ± SEM. **P < 0.01. (*n* = 3) **(D)** Representative images of immunocytochemical staining of ROS marker 3-NT in TKE2 (upper panel) and MCE (lower panel). (Scale bars: 80 μm.) **(E)** Representative images and analysis of western blot of 3-NT in TKE2. The blots were run under the same experimental conditions and the images were from the same gel. Data represent mean ± SEM. **P < 0.01. (*n* = 3) **(F)** Representative images and analysis of western blot of 3-NT in MCE. The blots were run under the same experimental conditions and the images were from the same gel. Data represent mean ± SEM. *P < 0.05. (*n* = 3) **(G)** SOD activity was measured by the SOD assay kit. SOD activity of TKE2. Data represent mean ± SEM. **P < 0.01. (*n* = 3) **(H)** SOD activity of MCE. Data represent mean ± SEM. ***P < 0.001. (*n* = 3).

We first applied H_2_O_2_-induced oxidative stress to cultured murine corneal epithelial progenitor cells (TKE2)^16^ and cultured mature murine corneal epithelial cells (MCE), and measured the cell viability by CCK-8 assay. The cell viability of MCE was significantly decreased in a concentration-dependent manner as the concentration of H_2_O_2_ increased, in contrast, TKE2 showed a substantial resistance to the cyto-toxicity induced by H_2_O_2_ (Fig. 1A).

To evaluate and compare the oxidative responses induced by H_2_O_2_ between these two types of cells, we examined the production of reactive oxygen species (ROS), the expression of oxidative stress marker 3-Nitrotyrosine (3-NT), and the activity of superoxide dismutases (SOD) which is a major scavenger of ROS. It was shown that H_2_O_2_ induced reductions of ROS production in TKE2 (Fig. 1B), while increases of ROS production in MCE (Fig. 1C). It was demonstrated by immunocytochemistry and western blot analysis that 3-NT levels in TKE2 were suppressed (Fig. 1D,E); on the other hand, 3-NT levels in MCE were increased (Fig. 1D,F). In addition, the activity of SOD was increased significantly in TKE2 (Fig. 1G), while decreasing in MCE after exposure to 0.25 mM H_2_O_2_ (Fig. 1H).

These results indicate that TKE2 cells have a stronger resistance and different responses against the oxidative stress induced by H_2_O_2,_ compared with those in MCE. Meanwhile we compared and analyzed the basic status of TKE2 and MCE by focusing on the different expressions of stem cell associated markers to verify that TKE2 cells have characteristics of stem/progenitor cells. As shown by Supplementary Fig. S1A,B, TKE2 showed higher levels of ATP-Binding Cassette Transporter G2 (ABCG2), an associated marker of adult stem cells, than that of MCE by immunochemical staining (Fig. S1A) and qRT-PCR assay (Fig. S1B). It was revealed that the expression level of another stem cell associated marker N-cadherin in TKE2 was higher than that in MCE as shown by immunostaining (Fig. S1C) and western blot analysis (Fig. S1D). In addition, the expression of PAX6, an indicator during the development of corneal epithelial cells, was significantly lower in TKE2, compared with that in MCE (Fig. S1E).

### The Alteration of ROS Generation Enzyme Induced by H_2_O_2_

**Figure 2:**
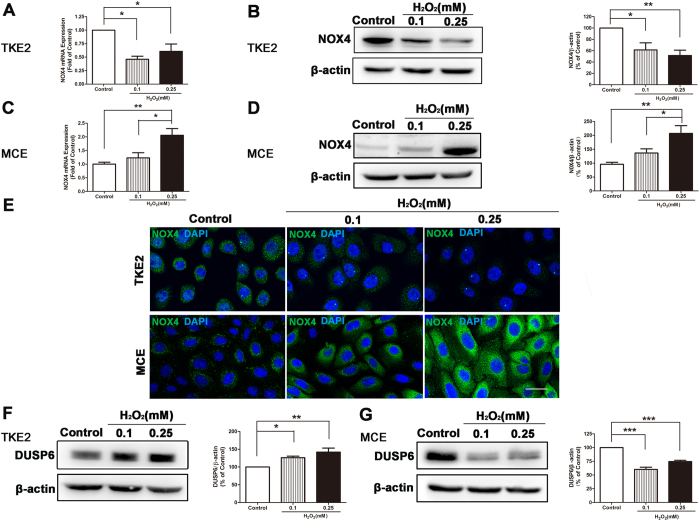
Alteration of NOX4 induced by H_2_O_2_. **(A)** qRT-PCR of NOX4 expression in TKE2. Data represent mean ± SEM. *P < 0.05. (*n* = 3) **(B)** Representative images and analysis of western blot of NOX4 in TKE2. The blots were run under the same experimental conditions and the images were from the same gel. Data represent mean ± SEM. *P < 0.05, **P < 0.01. (*n* = 5) **(C)** qRT-PCR of NOX4 expression in MCE. Data represent mean ± SEM. *P < 0.05, **P < 0.01 (*n* = 5). **(D)** Representative images and analysis of western blot of NOX4 in MCE. The blots were run under the same experimental conditions and the images were from the same gel. Data represent mean ± SEM. *P < 0.05, **P < 0.01 (*n* = 7). **(E)** Representative images of immunocytochemical staining of NOX4 in TKE2 (upper panel) and MCE (lower panel). (Scale bars: 80 μm). **(F)** Representative images and analysis of western blot of DUSP6 in TKE2. The blots were run under the same experimental conditions and the images were from the same gel. Data represent mean ± SEM. *P < 0.05, **P < 0.01. (*n* = 5) **(G)** Representative images and analysis of western blot of DUSP6 in MCE. The blots were run under the same experimental conditions and the images were from the same gel. Data represent mean ± SEM. ***P < 0.001. (*n* = 4).

To illustrate the underlying mechanism responsible for the different response to oxidative stress between TKE2 and MCE, we first assessed the expression of a key enzyme of ROS formation: NOX4 which is a major member of NADPH oxidase (NOX) family^20^,^21^. It was demonstrated by qRT-PCR and western blot analysis that NOX4 expression of TKE2 was inhibited by H_2_O_2_ (Fig. 2A) whereas NOX4 protein levels of TKE2 were also down-regulated after the treatment of H_2_O_2_ (Fig. 2B); on the other hand, both NOX4 expression and NOX4 protein levels of MCE were increased after exposure to H_2_O_2_ (Fig. 2C,D). Furthermore, immunocytochemistry staining also showed that NOX4 signal was down-regulated in TKE2 after exposure to H_2_O_2_; while intensified in MCE after treatment of H_2_O_2_ (Fig. 2E).

It is reported recently that the accumulation of intracellular H_2_O_2_ inactivates mitogen-activated protein kinase phosphatases (MKPs)^22^,^23^, and that dual specificity phosphatase 6 (DUSP6) is a member of the family of MKPs. We then conducted western blot analysis of DUSP6 between TKE2 and MCE to determine if the responses against the induction of H_2_O_2_ are via targeting DUSP6. Interestingly, it demonstrated that levels of DUSP6 were increased in TKE2 (Fig. 2F), in contrast, levels of DUSP6 were suppressed in MCE after treatment of H_2_O_2_ (Fig. 2G).

These data suggest that TKE2 cells have stronger resistance to H_2_O_2_ through inhibition of ROS generation enzyme.

### Activation of NRF2 Signaling Pathway Induced by H_2_O_2_

It is known that the NRF2 signaling pathway and its downstream factors, such as glutathione S-transferase (GSTP), NAD(P)H dehydrogenase (quinone 1) (NQO1), play vital roles in the degradation of ROS^24^,^25^. We then determined if the NRF2 signaling pathway was activated in these two types of cells after exposure to H_2_O_2_, by focusing on the expression levels of key factor NRF2 and downstream factor GSTP.

**Figure 3:**
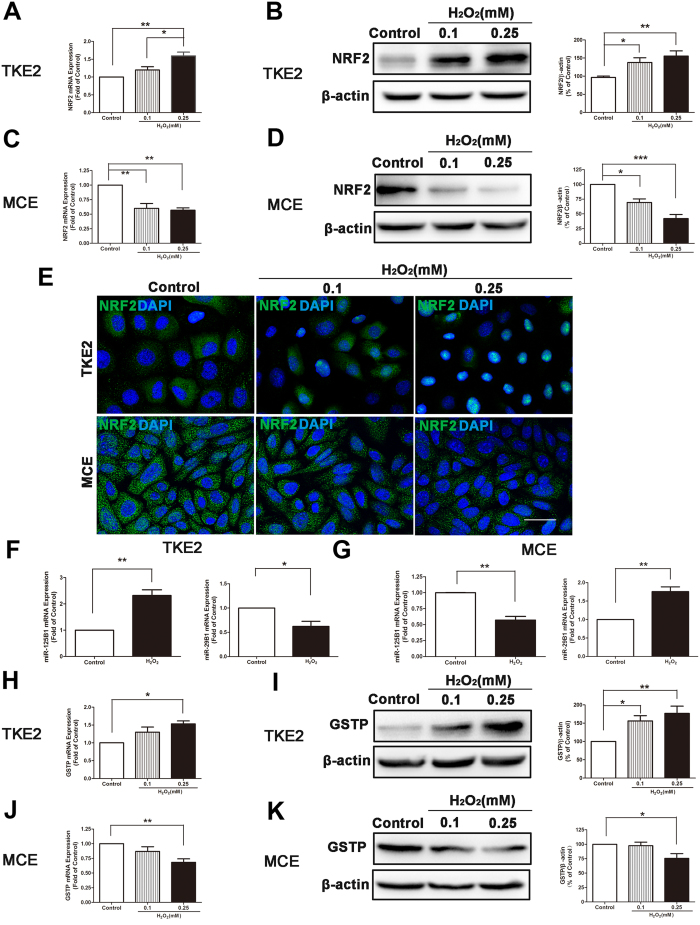
Activation of the NRF2 signaling pathway induced by H_2_O_2_. **(A)** qRT-PCR of NRF2 expression in TKE2. Data represent mean ± SEM. *P < 0.05, **P < 0.01. (*n* = 4) **(B)** Representative images and analysis of western blot of NRF2 in TKE2. The blots were run under the same experimental conditions and the images were from the same gel. Data represent mean ± SEM. *P < 0.05, **P < 0.01. (*n* = 8) **(C)** qRT-PCR of NRF2 expression in MCE. Data represent mean ± SEM. **P < 0.01. (*n* = 3) **(D)** Representative images and analysis of western blot of NRF2 in MCE. The blots were run under the same experimental conditions and the images were from the same gel. Data represent mean ± SEM. *P < 0.05, ***P < 0.001. (*n* = 3). **(E)** Representative images of immunocytochemical staining of NRF2 in TKE2 (upper panel) and MCE (lower panel) (Scale bars: 80 μm). **(F)** qRT-PCR of miR-125B1 and miR-29B1 inTKE2. Data represent mean ± SEM. *P < 0.05, **P < 0.01. (*n* = 3) **(G)** qRT-PCR of miR-125B1 and miR-29B1 in MCE. Data represent mean ± SEM. **P < 0.01. (*n* = 3–4) **(H)** qRT-PCR of GSTP expression inTKE2. Data represent mean ± SEM. *P < 0.05. (*n* = 3) **(I)** Representative images and analysis of western blot of GSTP in TKE2. The blots were run under the same experimental conditions and the images were from the same gel. Data represent mean ± SEM. *P < 0.05, **P < 0.01. (*n* = 4) **(J)** qRT-PCR of GSTP expression in MCE. Data represent mean ± SEM. **P < 0.01. (*n* = 4) **(K)** Representative images and analysis of western blot of GSTP in MCE. The blots were run under the same experimental conditions and the images were from the same gel. Data represent mean ± SEM. *P < 0.05 (*n* = 4).

It was shown that NRF2 expression of TKE2 was increased (Fig. 3A) whereas NRF2 protein levels of TKE2 were also up-regulated after the treatment of H_2_O_2_ (Fig. 3B). On the other hand, both NRF2 mRNA and protein levels of MCE were reduced after exposure to H_2_O_2_ (Fig. 3C,D). In addition, the immunocytochemistry staining results also showed that NRF2 signal of TKE2 was increased after induction of H_2_O_2_, compared with MCE. Furthermore, there were nuclear translocations of NRF2, an indicator of activation of the NRF2 signaling pathway, at higher concentration of H_2_O_2_ in TKE2 (Fig. 3E).

Multiple studies documented that miR-125B1 and miR-29B1 are associated with activation of the NRF2 pathway. NRF2 binds to the sites of antioxidant responsive element (ARE) in the promoters of miR-125B1 and miR-29B1. NRF2 functionally promotes the expression of miR125B1, while NRF2 negatively controls miR-29B1expression^26^,^27^. We next measured expression of both miR-125B1 and miR-29B1 in these two types of cells. Interestingly, exposure of 0.25 mM H_2_O_2_ up-regulated miR-125B1 expression, but down-regulated miR-29B1 expression in TKE2 (Fig. 3F). In contrast, the exposure to 0.25 mM H_2_O_2_ inhibited miR-125B1 expression, but increased miR-29B1 expression in MCE (Fig. 3G).

We next investigated the expression differences of the key downstream factor of the NRF2 signaling pathway GSTP between TKE2 and MCE. It was demonstrated that GSTP expression was elevated at the mRNA level (Fig. 3H) and protein level in TKE2 after exposure to H_2_O_2_ (Fig. 3I). In contrast, both GSTP mRNA and protein levels were down-regulated in MCE after exposure to H_2_O_2_ (Fig. 3J,k).

Taken together, these results suggest that the NRF2 signaling pathway and its downstream factors are activated after exposure to H_2_O_2_ in TKE2.

### Evidence of RNA Interference and Pharmacological Antagonization

**Figure 4:**
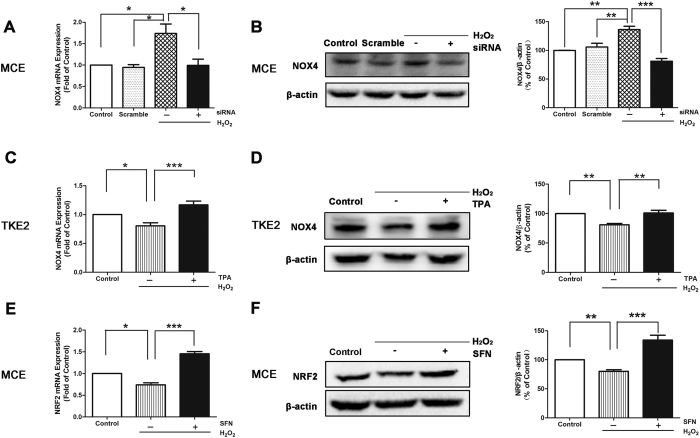
RNA Interference and Pharmacological Antagonization. **(A)** qRT-PCR of NOX4 expression in MCE after transfection of siRNA for NOX4. Data represent mean ± SEM. *P < 0.05. (*n* = 3) **(B)** Representative images and analysis of western blot of NOX4 in MCE after transfection of siRNA for NOX4. The blots were run under the same experimental conditions and the images were from the same gel. Data represent mean ± SEM. **P < 0.01, ***P < 0.001 (*n* = 4). **(C)** qRT-PCR of NOX4 expression in TKE2 after the treatment of the NOX4 agonist TPA. Data represent mean ± SEM. *P < 0.05, ***P < 0.001. (*n* = 5) **(D)** Representative images and analysis of western blot of NOX4 in TKE2 after the treatment with the TPA. The blots were run under the same experimental conditions and the images were from the same gel. Data represent mean ± SEM. **P < 0.01. (*n* = 4) **(E)** qRT-PCR of NRF2 expression in MCE after the treatment with the NRF2 agonist SFN. Data represent mean ± SEM. *P < 0.05, ***P < 0.001. (*n* = 3) **(F)** Representative images and analysis of western blot of NOX4 in MCE after the treatment of SFN. The blots were run under the same experimental conditions and the images were from the same gel. Data represent mean ± SEM. **P < 0.01, ***P < 0.001. (*n* = 4).

To further confirm the up-regulation of NOX4 induced by H_2_O_2_, we knocked down the gene expression of NOX4 in MCE with RNA interference approach. It was demonstrated by qRT-PCR assay and western blot analysis that NOX4 si-RNA antagonized the increases of NOX4 expression at both the mRNA level (Fig. 4A) and protein levels in MCE after exposure to 0.25 mM H_2_O_2_ (Fig. 4B).

It was also revealed that NOX4 expression was inhibited in TKE2 cells after treatment of H_2_O_2_. We then applied a NOX4 agonist 12-o-tereadecanoylphorbol-13-acetate (TPA) in TKE2 to antagonize the inhibited level of NOX4. The qRT-PCR assay results showed that TPA reversed the down-regulation of NOX4 expression induced by 0.25 mM H_2_O_2_ (Fig. 4C), whereas TPA elevated the NOX4 protein level in TKE2 cells treated by 0.25 mM H_2_O_2_ (Fig. 4D).

On the other hand, our results showed that NRF2 expression was suppressed by H_2_O_2_ in MCE. We next administered a NRF2 agonist Sulforaphane (SFN) to antagonize the effect of H_2_O_2_ on NRF2 in MCE. It was revealed that the suppressed NRF2 expression induced by 0.25 mM H_2_O_2_ was reversed after the SFN treatment (Fig. 4E). Furthermore, western blotting results also showed that the suppressed NRF2 protein levels induced by 0.25 mM H_2_O_2_ were reversed by the SFN treatment (Fig. 4F).

These results of RNA interference and pharmacological antagonization further supported and confirmed that the mechanism responsible for the different responses to oxidative stress between TKE2 and MCE are through targeting NOX4 and NRF2.

### Possible Association with Autophagy

**Figure 5:**
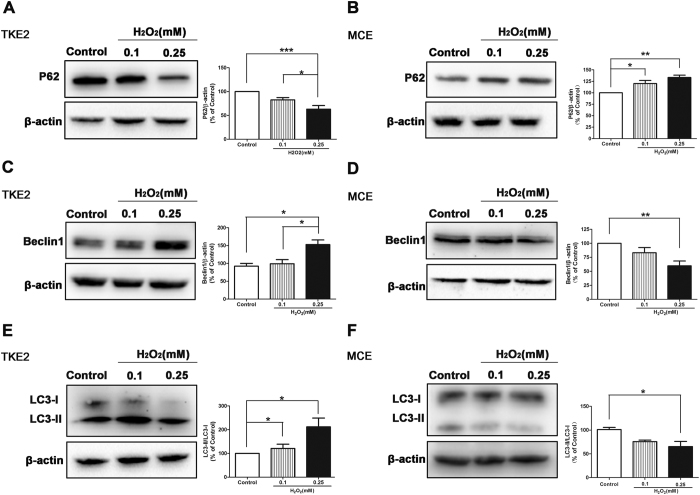
Alterations of autophagy markers. **(A)** Representative images and analysis of western blot of P62 in TKE2. The blots were run under the same experimental conditions and the images were from the same gel. Data represent mean ± SEM. *P < 0.05, ***P < 0.001.(*n* = 6) **(B)** Representative images and analysis of western blot of P62 in MCE. The blots were run under the same experimental conditions and the images were from the same gel. Data represent mean ± SEM. *P < 0.05, **P < 0.01. (*n* = 4) **(C)** Representative images and analysis of western blot of Beclin1 in TKE2. The blots were run under the same experimental conditions and the images were from the same gel. Data represent mean ± SEM. *P < 0.05. (*n* = 3) **(D)** Representative images and analysis of western blot of Beclin1 in MCE. Data represent mean ± SEM. **P < 0.01. (*n* = 5) **(E)** Representative images and analysis of western blot of the ratio of LC3-II and LC3-I in TKE2. The blots were run under the same experimental conditions and the images were from the same gel. Data represent mean ± SEM. *P < 0.05. (*n* = 5) **(F)** Representative images and analysis of western blot of the ratio of LC3-II and LC3-I in MCE. The blots were run under the same experimental conditions and the images were from the same gel. Data represent mean ± SEM.*P < 0.05 (*n* = 3).

Association between ROS and process of autophagy has drawn increasing attention recently. It has been reported that ROS induces autophagy^28^,^29^; on the other hand, autophagy may reduce oxidative damage^30^,^31^,^32^,^33^. In the present study, we investigated the different levels of key factors of autophagy, such as P62, Beclin1, and LC3-I and LC3-II, in TKE2 and MCE after treatment of H_2_O_2_ for 4 hours. It was demonstrated by western blot analysis that protein levels of P62, which is known as a linker molecule between ubiquitinated proteins and the autophagy system, was suppressed in TKE2 (Fig. 5A), but up-regulated in MCE after exposure to H_2_O_2_ (Fig. 5B). Furthermore, levels of another important factor of autophagy, Beclin1, were up-regulated in TKE2 after exposure to H_2_O_2_ (Fig. 5C), but were decreased in MCE (Fig. 5D). In addition, it is well known that, along with autophagy activation, the LC3-I protein is converted into the LC3-II form which is specifically associated with the autophagosomal membrane. Therefore, the amount of LC3-II, or the ratio of LC3-II and LC3-I, has served as a good indicator of the autophagic flux. The ratio of LC3- II and LC3-I was increased in TKE2 (Fig. 5E), but decreased in MCE after the treatment of H_2_O_2_ (Fig. 5F). These results indicate that the stronger resistance to oxidative stress of TKE2 cells might be caused by the enhancement of cellular autophagy.

### Comparisons of Differentiated States of TKE2

To further determine if the distinct responses and mechanisms underlying oxidative stress is unique only in the state of stem cell or progenitor cells, but not in differentiated or mature cells, we conducted a series of studies to compare and analyze two states of TKE2: 1) stem cell like state that we applied in this study and demonstrated markers of stem cells, and 2) the differentiated or mature TKE2 which was transformed from stem cell like state by a different culture condition.

**Figure 6:**
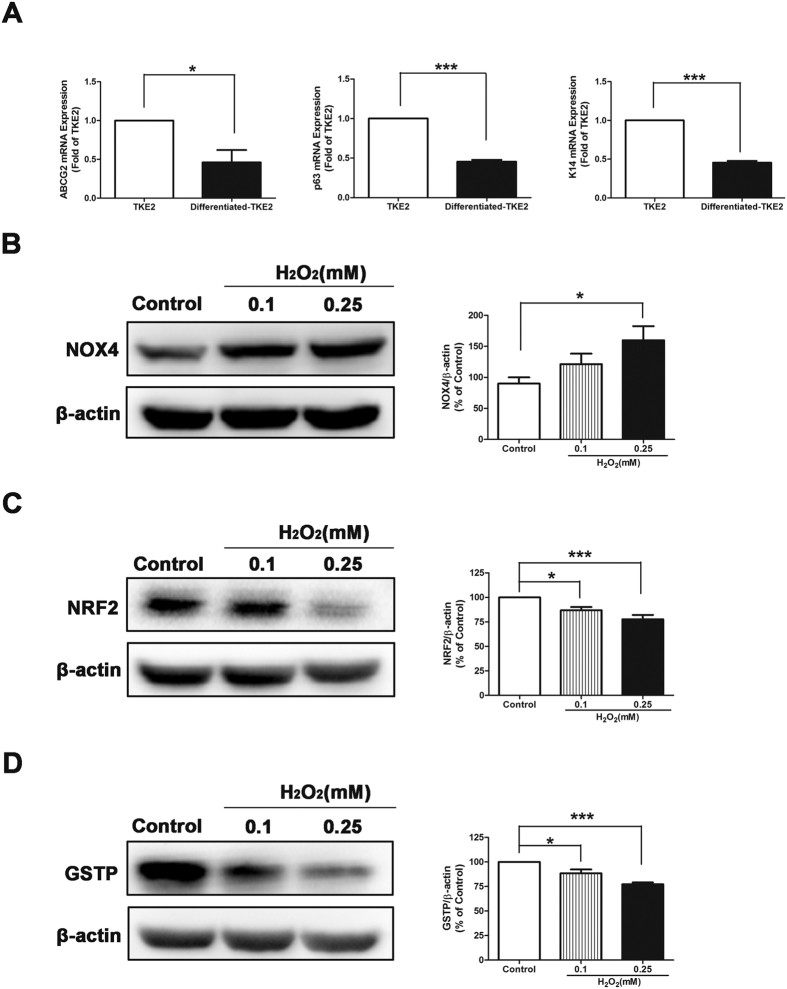
Comparisons of Differentiated States of TKE2. **(A)** qRT-PCR analysis of ABCG2, P63 and K14 expression between TKE2 and differentiated TKE2. Data represent mean ± SEM. *P < 0.05, ***P < 0.001. (ABCG2: *n* = 4; P63: *n* = 4; K14: *n* = 4) **(B)** Representative images and analysis of western blot of NOX4 in differentiated TKE2. The blots were run under the same experimental conditions and the images were from the same gel. Data represent mean ± SEM. *P < 0.05. (*n* = 5) **(C)** Representative images and analysis of western blot of NRF2 in differentiated TKE2. The blots were run under the same experimental conditions and the images were from the same gel. Data represent mean ± SEM. *P < 0.05, ***P < 0.001. (*n* = 6) **(D)** Representative images and analysis of western blot of GSTP in differentiated TKE2. The blots were run under the same experimental conditions and the images were from the same gel. Data represent mean ± SEM. *P < 0.05, ***P < 0.001. (*n* = 8).

We first assessed and compared the different gene expressions of stem cell associated markers ABCG2, Tumor Protein P63 (P63) and Keratin 14(K14) between two states of TKE2. As shown by qRT-PCR, mRNA levels of ABCG2, P63 and K14 were down-regulated in the differentiated TKE2 (Fig. 6A), suggesting that TKE2 maintain the cell phenotypes of stem cells or progenitor cells.

As shown by western blot analysis, NOX4 protein levels were up-regulated after treatment of H_2_O_2_ in differentiated TKE2 (Fig. 6B), meanwhile protein levels of NRF2 and GSTP were decreased after exposure to H_2_O_2_ in differentiated TKE2 (Fig. 6C,D). These results of differentiated TKE2 were similar to those of MCE. It indicates that the unique antioxidant property only exist in the stem cell or progenitor cell state of TKE2, but not in the differentiated state of TKE2.

## Discussion

We provide novel evidence that murine corneal epithelial progenitor cell demonstrates a different response to oxidative stress, compared with mature or differentiated murine corneal epithelial cell. Murine corneal epithelial progenitor cell shows a more potent antioxidant cell property due to a different pattern of the redox system and signaling pathway, including suppression of ROS formation, activation of the NRF2 signaling pathway and increases of antioxidants. These observations will contribute to the better understanding of the role of ROS in stem cell biology. The results also indicate potential beneficial values of corneal stem/progenitor cells in therapy, transplantation and other applications.

The redox balance is a main cellular biochemical process, and a redox imbalance may result in excessive production of ROS which may lead to oxidative stress. ROS homeostasis is regulated by ROS generation and degradation. ROS is formed mainly in mitochondria, and NOX4 is a key enzyme of ROS formation^34^. Many traditional antioxidants are scavengers of ROS products^35^. Multiple reports demonstrate that the balance between self-renewal and differentiation of stem cells is partly regulated by ROS, and stem cells have antioxidant properties^4^,^5^,^6^,^7^. Our results support the stronger antioxidant property of stem cells. However, the role of ROS in stem cell biology remains largely unknown. For example, it remains elusive whether there is a unique redox homeostasis or balance in stem cells, how ROS affects cell division and differentiation and other functions of stem cells, and what is the role of ROS after stem cell transplantation and in other stem cell applications.

In this present study, we demonstrate that murine corneal epithelial progenitor cells, unlike mature or differentiated murine corneal epithelial cells, have distinct homeostasis under oxidative stress or have stronger antioxidant properties. We also show that the key factors of the ROS system in these two cells respond differently against oxidative stress. One hypothesis is that redox homeostasis or balance in corneal epithelial progenitor cells are changed or modified during the process of cell division and differentiation. Another possibility might be that an yet to be identified system, target or “switch”, which controls and regulates the key factors in ROS generation, is turned on/off or adjusted between corneal epithelial progenitor cells and mature and differentiated corneal epithelial cells. Meanwhile, we also indentified and briefly compared the basic phenotypes of murine corneal epithelial progenitor cell and mature murine corneal epithelial cell in the absence of oxidative stress. Further quantitative analysis of ROS products and more investigation on the key factors of ROS are needed to compare the basic states between these two cells in normal condition.

The NRF2 signaling pathway is believed to be a major ROS signaling pathway. Activation of the NRF2 signaling pathway and its downstream factors, such as GSTP and NQO1, plays a vital role in the antioxidant activities^36^,^37^. A recent study suggests that the activation of the endogenous NRF2 signaling pathway and the subsequent increases of antioxidant responsive element (ARE) members is an efficient and new approach for the exploration of next generation of antioxidants, which are different from the traditional and classic small molecule antioxidants which have potential side effects in clinical application^34^. Our novel experimental evidence reveals that murine corneal epithelial progenitor cell activates the NRF2 signaling pathway and induces increases of antioxidant levels under oxidative stress, indicating a potential therapeutic value of corneal epithelial stem/progenitor cells transplantation.

It is believed that autophagy is associated with the oxidative stress of cells^38^,^39^,^40^. When excessive ROS is generated, these ROS products will lead to the process of autophagy, on the other hand, a major function of autophagy helps to remove the damaged cellular organelles and to promote the survival of cells^41^,^42^. Interestingly, murine corneal epithelial progenitor cells induce autophagy at early stage under oxidative stress in our experiments. In contrast, the autophagy is inhibited in mature murine corneal epithelial cells. It suggests that the stronger antioxidant activity of corneal epithelial progenitor cell is, at least partially, associated with the early increases of key factors of autophagy, although further investigation on the relation between ROS and autophagy in corneal stem cells is required.

We examined the different responses to oxidative stress between two states of murine corneal epithelial progenitor cells, TKE2 cells. The TKE2 in stem cell status, unlike the differentiated or mature TKE2, have stronger antioxidant properties under oxidative stress, indicating that redox homeostasis alters from the stem cell state and differentiated state. In other words, the ROS balance is changed or adjusted after corneal epithelial progenitor cells transform into differentiated or terminal corneal epithelial cells under oxidative stress. More detailed information or experimental evidence about key factors of ROS in the basic state, i.e., in the absence of oxidative stress, between these two states of TKE2 will help to better understand the transition of the ROS system and pathway during cell division and differentiation of corneal epithelial progenitor cells.

Illustration of the role of ROS in stem cell biology will help to better understand the unique complex cellular functions and networks of stem cells, the process of cell division and differentiation of stem cells as well as the potential values in the application and transplantation of stem cells in the future.

## Additional Information

**How to cite this article**: Zhou, J. *et al*. ROS-mediated Different Homeostasis of Murine Corneal Epithelial Progenitor Cell Line under Oxidative Stress. *Sci. Rep.* **6**, 36481; doi: 10.1038/srep36481 (2016).

**Publisher’s note**: Springer Nature remains neutral with regard to jurisdictional claims in published maps and institutional affiliations.

## Supplementary Material

Supplementary Information
