# Supplementary material for: ROS-mediated Different Homeostasis of Murine Corneal Epithelial Progenitor Cell Line under Oxidative Stress
Source: Sci Rep. 2016 Nov 2;6:36481. doi: 10.1038/srep36481 (PMC5090348; doi:10.1038/srep36481)
Supplement: Supplementary Information [file srep36481-s1.pdf]

Supplementary data

ROS-mediated Different Homeostasis of Murine Corneal Epithelial Progenitor Cell Line under Oxidative Stress

Jing Zhou[1#], Lianping Ge[1#], Changkai Jia[1], Xiling Zheng[1], Huixia Cui[1], Rongrong Zong[1], Xiaorui Bao[1], Yuanyuan Yin[1], Jian-xing Ma[1], Wei Li[1], Zuguo Liu[1], Yueping Zhou[1,*]

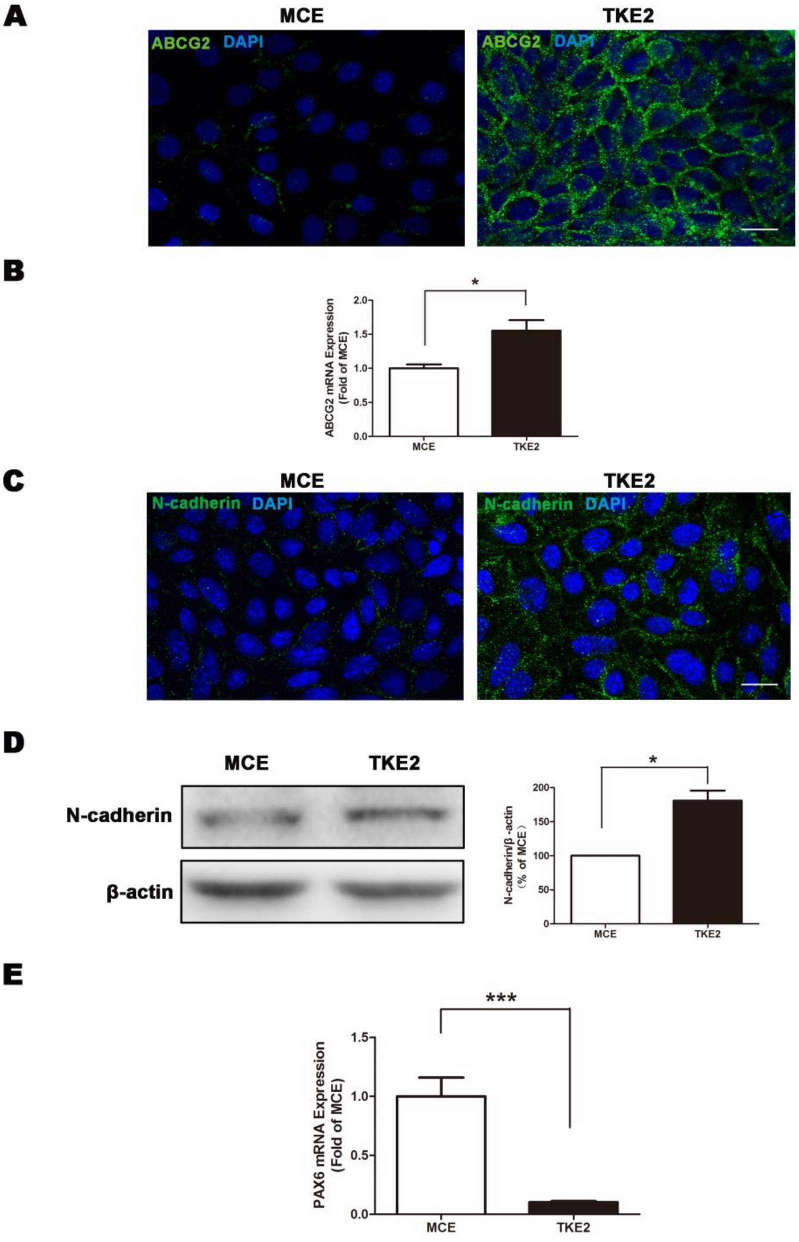

Supplementary Figure S1. Phenotypes of TKE2 and MCE. (A) Representative images of immunocytochemical staining of ABCG2 (Scale bars: 80 μm). **(B)** Quantitative real-time PCR (qRT-PCR) of ABCG2 expression. Data represent mean ± SEM. *$P < 0.05$. ($n = 3$) **(C)** Representative images of immunocytochemical staining of N-cadherin (Scale bars: 80 μm). **(D)** Representative images and analysis of western blot of N-cadherin. The blots were run under the same experimental conditions and the images were from the same gel. Data represent mean ± SEM. *$P < 0.05$. ($n = 4$) **(E)** qRT-PCR analysis of PAX6 expression. Data represent mean ± SEM. ***$P < 0.001$. ($n = 6$)

Supplementary Table S1. Primers used for quantitative RT-PCR

Gene	Forward (5' to 3')	Reverse (5' to 3')
NRF2	CCCAGCAGGACATGGATTTGA	AGCTCATAGTCCTTCTGTCGC
GSTP	TCTACGCAGCACTGAATCCG	GGAGCTGCCCATACAGACAA
NOX4	GTGCGGAGAGACTTTACCGA	ACTGGCCAGGTCTTGCTTTA
ABCG2	CCTCACCTTACTGGCTTCCG	ATCCGCAGGGTTGTTGTAGG
P63	ATGTCACCGAGGTTGTGAAA	GAATTCAGTGCCAACCTGTG
K14	CCCACCTTTCATCTTCCCAATT	AAGCCTGAGCAGCATGTAGCAG
PAX6	AGTGTCTACCAGCCAATCCC	CATGGAACCTGATGTGAAGG
β-actin	CCTCTATGCCAACACAGTGC	CCTGCTTGCTGATCCACATC
